# CO-DESIGNING DATA COLLECTION AND USE PROTOCOLS FOR PROVIDING PERSON-CENTERED DEMENTIA CARE

**DOI:** 10.1093/geroni/igae098.0573

**Published:** 2024-12-31

**Authors:** Kirsten Corazzini, Eleanor McConnell, Nancy Kusmaul, Sarah Holmes, Jing Wang

**Affiliations:** University of New Hampshire, Durham, New Hampshire, United States; Duke University, Durham, North Carolina, United States; University of Maryland Baltimore County, Baltimore, Maryland, United States; University of Maryland School of Nursing, Baltimore, Maryland, United States; University of New Hampshire, Durham, New Hampshire, United States

## Abstract

Considerable gaps remain in delivering person-centered dementia care in residential long-term care (LTC) settings, despite gains in instrument development, and the addition of person-centered care-related items in the Minimum Data Set (MDS). The paucity of pragmatic measures and care processes to support person-centered dementia LTC has been identified as significantly contributing to this gap. Further, this gap is exacerbated in medically underserved and health professional shortage (MUHPS) communities, which are less likely to have the professional development and technical capacity to support robust data collection and use of person-centered care measures among front-line care staff. This multi-method, participatory research study aimed to address this gap by co-designing, with residential long-term care residents who are living with dementia, their families, and care staff in MUHPS communities, pragmatic data collection and use protocols. This paper describes the development of co-design workshops through the data analysis of qualitative interviews (N=68 residents, family members, and staff from 4 LTC communities), and results from co-design workshops of identified strategies following review of journey maps (N=8 workshops). Results provided low-resource strategies in multiple domains over time for staff members (onboarding, developing expertise, and becoming an expert); residents (moving into your new home, being who you are and living, and feeling like you are settled); and families (supporting a family member’s move, supporting good care, and overall assessment of care). Findings advance our methodological and substantive capacity for inclusive and low-resource strategies to improve person-centered dementia care practice in LTC.

